# Magnetic hydrogels with ordered structure for biomedical applications

**DOI:** 10.3389/fchem.2022.1040492

**Published:** 2022-10-11

**Authors:** Le Xue, Jianfei Sun

**Affiliations:** State Key Laboratory of Bioelectronics, Jiangsu Key Laboratory of Biomaterials and Devices, School of Biological Science and Medical Engineering, Southeast University, Nanjing, China

**Keywords:** ordered structure, magnetic nanoparticles, magnetic hydrogels, magnetic field, biomedical applications

## Abstract

Magnetic hydrogels composed of hydrogel matrices and magnetic nanomaterials have attracted widespread interests. Thereinto, magnetic hydrogels with ordered structure possessing enhanced functionalities and unique architectures, show tremendous advantages in biomedical fields. The ordered structure brought unique anisotropic properties and excellent physical properties. Furthermore, the anisotropic properties of magnetic ordered hydrogels are more analogous to biological tissues in morphology and mechanical property, showing better biocompatibility and bioinducibility. Thus, we aim to systematically describe the latest advances of magnetic hydrogels with ordered structure. Firstly, this review introduced the synthetic methods of magnetic hydrogels focus on constructing ordered structure. Then, their functionalities and biomedical applications are also summarized. Finally, the current challenges and a compelling perspective outlook of magnetic ordered hydrogel are present.

## Introduction

Hydrogels are cross-linked polymeric network containing substantial amounts of water, which is similar to the native extracellular matrix (Daly et al., 2020). Owing to their good hydrophilicity, elasticity, biocompatibility and degradability, hydrogels have been widely used in biomedical and pharmaceutical fields such as controlled drug delivery, biosensing, cell culturing and tissue engineering (Seliktar, 2012; Liu et al., 2020; Shi et al., 2020a; Kailasa et al., 2022; Wang et al., 2022a; Yarali et al., 2022). Despite the purported advantages of conventional hydrogels and burgeoning efforts to utilize them in biomedical applications, they are usually trapped by limited functionality and poor controllability (Lee et al., 2013; Shi et al., 2020a; Ge et al., 2021). Therefore, a growing attention is concentrated on imparting functionality to hydrogels. The functional hydrogels (also referred to as smart hydrogels) obtained optimized structure, enhanced performance and environmentally specific controllability, which greatly extends the application in many areas (Xia et al., 2013; Sikdar et al., 2021; Bordbar-Khiabani and Gasik, 2022; Sun et al., 2022).

In general, functional hydrogels are capable to response remotely to external physical stimuli such as heat, light, force, electric fields and magnetic fields (Koetting et al., 2015; Fu et al., 2018; Vazquez-Gonzalez and Willner, 2020; Li et al., 2021; Kailasa et al., 2022). Among various types of functional hydrogels, magnetic hydrogels have attracted extensive researches and achieved remarkable progress in terms of fabrication and biomedical applications (Hu et al., 2015a; Shi et al., 2020a). Magnetic hydrogels usually combine hydrogel networks with magnetic nanoparticles, making they sensitive to external magnetic field (Shi et al., 2020a). While in biomedical applications, the magnetic field can be remotely applied and induce rapid and accurate response (Kondaveeti et al., 2018; Tang et al., 2021). In addition, it is a relatively mild stimulus, avoiding adverse effects on biological soft tissue (Uva et al., 2015; Cruz et al., 2021). Conventional magnetic hydrogels usually exhibit homogeneous structure which means that magnetic nanoparticles are all randomly distributed throughout the hydrogel without order (Mun et al., 2020; Pardo et al., 2021). However, most homogeneous magnetic hydrogels continue to face challenges with practical applications because of the limited performance and incoordination with tissues (Shi et al., 2020a; Li K. et al., 2022).

Many biological tissues such as eye, muscle, skin and cartilage also present well-defined hierarchical, anisotropic structures (Zhang et al., 2016a; Shi et al., 2020b; Park and Kim, 2020; Sun et al., 2020). The intrinsic ordered structure of tissues plays an essential role in physiological functions and activities, such as muscle contraction and cell migration (Grazi, 2008; Zhukova et al., 2017). Thus, many efforts have been made to construct bionic structure (such as alignment, orientation and hierarchy) in materials, in order to achieve enhanced physical properties and good biocompatibility (Shi et al., 2020a; Reid et al., 2021; Xing et al., 2022). Magnetic anisotropic hydrogels have been created by utilizing magnetic nanoparticles to construct ordered structures (Araujo-Custodio et al., 2019; Bin et al., 2021; Chen et al., 2022a). Different from the isotropic networks of conventional hydrogels, the magnetic nanoparticles are orderly distributed inside the hydrogels, showing magnetic anisotropy as well as good controllability. Moreover, magnetic anisotropic hydrogels can simulate biological tissues better in morphology and mechanic property (Sano et al., 2018). In particular, the anisotropic structures also bring enhanced functionalities to magnetic hydrogels, such as more efficient drug delivery and thermogenesis under the magnetic field (Hu et al., 2015a; Bozuyuk et al., 2018). Therefore, many researches pay attention to the fabrication of magnetic hydrogels with ordered structure and the expansion of their biomedical applications (Araujo-Custodio et al., 2019; Yook et al., 2019; Chen et al., 2022b; Li K. et al., 2022).

**FIGURE 1:**
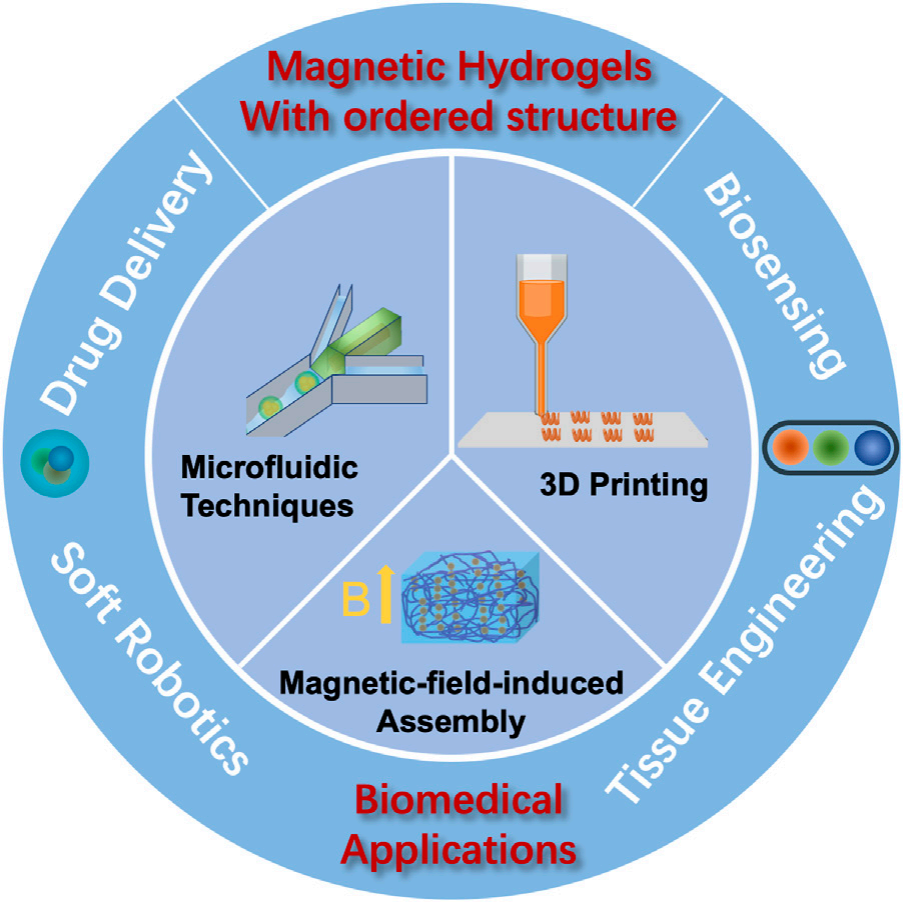
Schematic illustration of the fabrication of magnetic hydrogels with ordered structure and their biomedical applications.

Due to the practical value of magnetic hydrogels, there have been several reviews on certain aspects (Zhang et al., 2016b; Ganguly and Margel, 2021; Li et al., 2021). However, fewer investigations were concentrated on magnetic hydrogels with ordered structure. Herein, we aim to review the research progress of magnetic hydrogels with ordered structure, covering aspects ranging from the strategies of structure construction to biomedical applications (Figure 1). Finally, the challenges and outlook of magnetic anisotropic hydrogels for biomedical applications are presented.

## Construction of magnetic hydrogels with ordered structure

In general, magnetic hydrogels are composed of a hydrogel matrix and a magnetic component. A variety of magnetic nanomaterials have been incorporated into hydrogel networks, such as γ-Fe_2_O_3_, Fe_3_O_4_, and transition metal ferrite nanoparticles (CoFe_2_O_4_, MnFe_2_O_4_, etc.) (Lima-Tenorio et al., 2015; Naderi and Azizian, 2018; Ganguly and Margel, 2021). Among these magnetic materials, superparamagnetic iron oxide nanoparticles (γ-Fe_2_O_3_, Fe_3_O_4_) were the most promising candidate for clinical applications due to their good chemical stability, high magnetization ability and great biocompatibility (Meenach et al., 2009; Ling and Hyeon, 2013; Bustamante-Torres et al., 2022).

**FIGURE 2:**
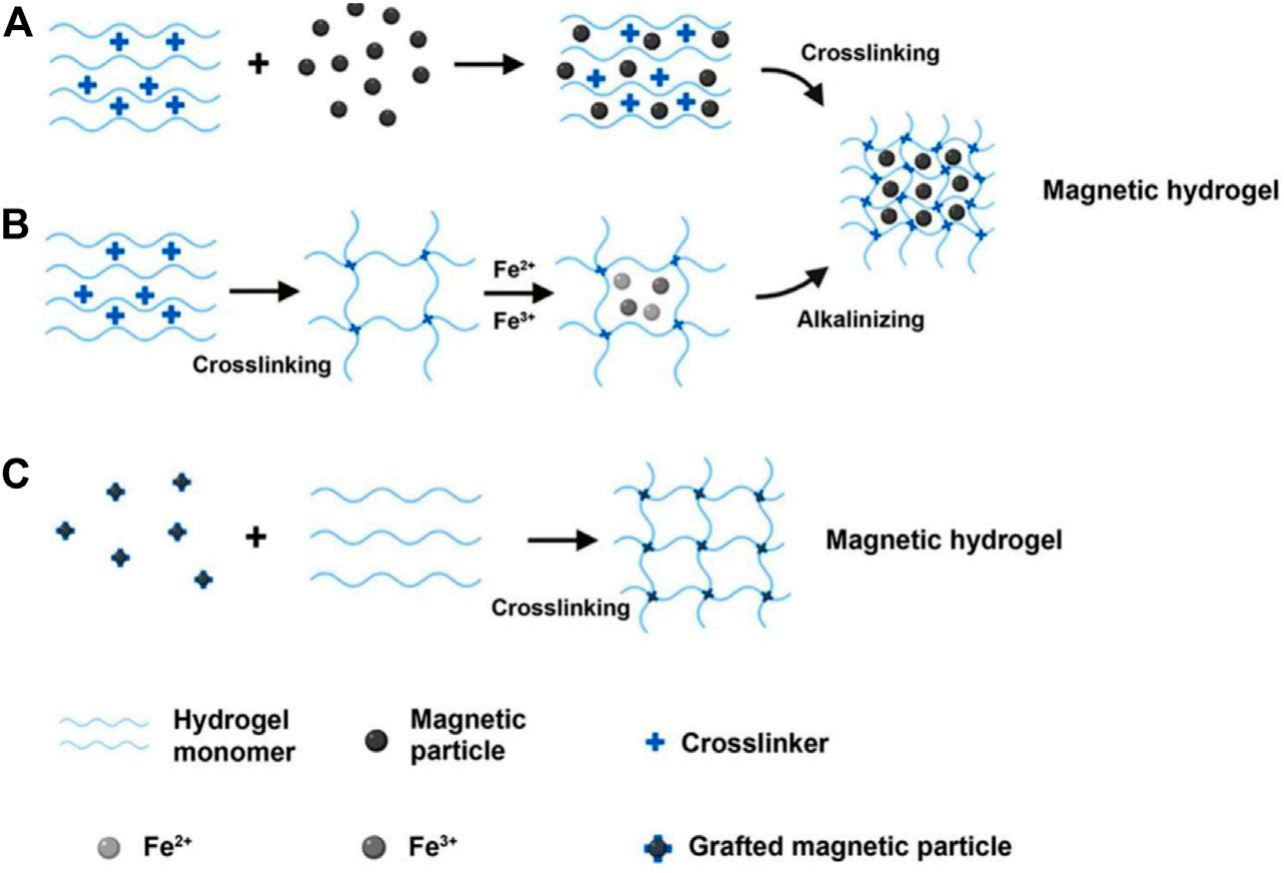
The schematic diagram of the main strategies for fabrication of magnetic hydrogels. **(A)** The blending method. **(B)** The precipitation method *in situ*. **(C)** The grafting-onto method [Reproduced with permission from (Liu et al., 2020) Copyright 2020 Frontiers].

Many endeavors have been conducted to fix magnetic materials inside polymer networks (Zhang et al., 2016b; Li et al., 2021). As systematically reviewed previously, the main strategies for fabrication of magnetic hydrogels were developed including the blending method, *in situ* precipitation method and the grafting-onto method, as shown in Figure 2 (Liu et al., 2020; Ganguly and Margel, 2021). Besides altering the type of hydrogel matrices and magnetic nanoparticles, the properties of magnetic hydrogels can also be readily modulated by the concentration, size and distribution of magnetic particles within the hydrogels (Li et al., 2013; Ganguly and Margel, 2021). Many biological tissues exhibit well-defined ordered structures, thus introducing ordered structures into hydrogel is necessary and significant which can mimic biological tissues more better and enhance performance (Liu et al., 2015). A variety of fabrication strategies have been developed to fabricate magnetic hydrogels with ordered structure (Sano et al., 2018; Li K. et al., 2022). We can categorize them as magnetic-field-induced assembly, microfluidics and 3D printing (Table 1). Magnetic-field-induced assembly is a controllable and easy method for construction of magnetic ordered materials by arrangement of magnetic nanoparticles under magnetic field (Shi et al., 2020a). Here, orderly manufacturing-based construction is another strategy, including microfluidics and three-dimensional (3D) printing (Daly et al., 2020; Cai et al., 2021; Yang et al., 2022b). This strategy offers the ability to prepare microscale hydrogels and unique control over the fabrication of complex structures (Zhang et al., 2021). In addition, magnetic-field-induced assembly can be integrated into 3D printing to fabricate anisotropic composites (Wang et al., 2017). In this section, we focus on how to introduce magnetic nanoparticles into the hydrogel matrices with ordered structure, and meanwhile illustrating the properties of magnetic hydrogels.

### Magnetic-field-induced assembly

Due to fast and reversible magnetic responsiveness of magnetic nanoparticles, magnetic-field-induced assembly can readily construct magnetic materials with ordered structure (Shi et al., 2020a). Commonly, ferromagnetic nanoparticles, paramagnetic nanoparticles and superparamagnetic nanoparticles can be could be obtained by fixing the magnetic assembly within their networks. In most cases, magnetostatic field was applied to form chain-like or column-like structure within hydrogels (Lu et al., 2012; Hu et al., 2015a; Hu et al., 2015b). Due to the magnetic dipolar interaction, the particles tend to form one-dimensional (1D) assembled structures quickly with long-range chain parallel to the imposed magnetic field (Shi et al., 2020a). Besides the strength of applied magnetic field, the morphology of the aligned magnetic nanoparticles chains can also be modulated by the concentration and size of magnetic nanoparticles (Zhang et al., 2021). Magnetic hydrogels with assembled structures exhibited enhanced functionality and controllability, showing more promising potentials in biomedical applications.

**FIGURE 3:**
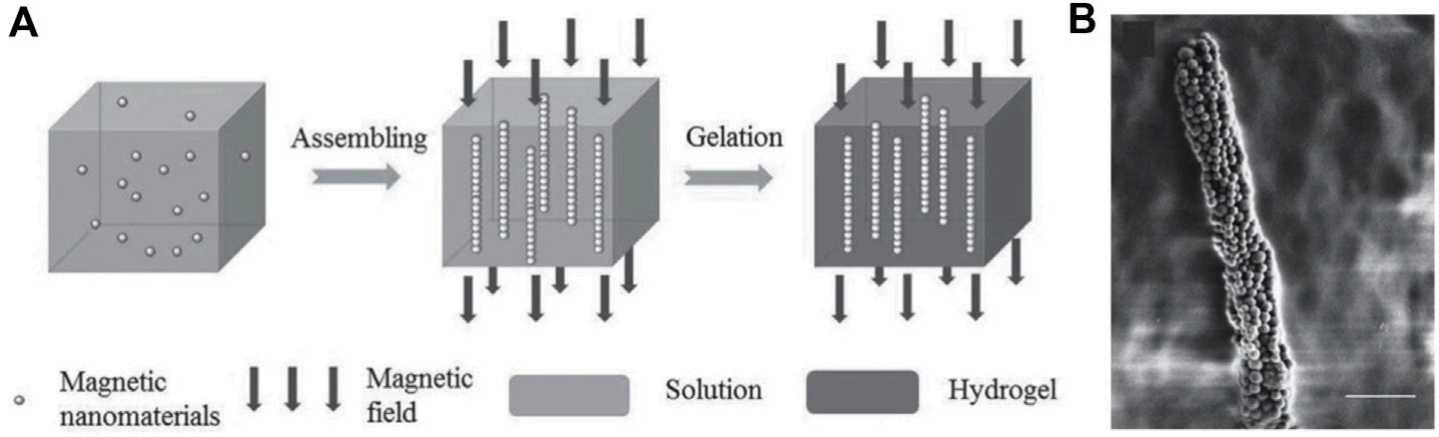
Morphology of the fabricated chain-like assemblies inside hydrogel. **(A)** Schematic diagram of the fabrication process for aligned magnetic hydrogel. **(B)** SEM of magnetic hydrogel with chains. Scale bar = 1 µm. (Reproduced with permission from (Hu et al., 2015a) Copyright 2015 Wiley-VCH).

The magnetothermal behavior of magnetic hydrogels can be enhanced and controlled by magnetic field of magnetic nanoparticles. In our previous work, we fabricated magnetic hydrogel by mixing the magnetic nanoparticles with monomers solution and gelating it in the presence of a magnetostatic field (Hu et al., 2015a). As shown in Figure 3, The magnetic nanoparticles formed chain-like assemblies inside the hydrogels because of the magnetic dipolar interaction. As the process of gelation was activated by heat without stirring, the morphology of assemblies could be kept perfectly. The magnetothermal effect was found to be enhanced when the direction of the external magnetic field was parallel to the constructed chains. Very recently, Rincon-Iglesias et al. (2022) prepared Fe_3_O_4_ nanorods coated with gold shell and oriented them inside agarose hydrogel by magnetic field. The nanorods formed linear assemblies, resulting in anisotropic magneto- and photo-thermia behavior at the same time. The synergistic thermal therapy meant less use of nanoparticles, which is benefit to biomedical applications. More interestingly, we found that using the rotation magnetic field could induce the disk-like assembly of magnetic nanoparticles inside the polyacrylamide hydrogel (Fan et al., 2018). Besides the dipolar forces, the rotation magnetic field could generate torques to induce the rotational motion of aligned magnetic assemblies, forming disk-like assemblies finally. And as the size of the microstructure increased, the magnetothermal anisotropy enhanced. The adjustable magnetothermal behavior of magnetic hydrogels showed promising potentials in the biomedical applications. In a recent work, a rotating magnetic field was used to orient two-dimensional (2D) magnetic double stacks (MDSs) which were composed of γ-Fe_2_O_3_ nanoparticles sandwiched by two silicate nanosheets, and then this unique structures were fixed inside the poly (N-isopropylacrylamide) hydrogel by polymerization (Dai et al., 2022). Based on the highly-ordered arrangement of MDSs, the hydrogels showed anisotropic optical, mechanical properties, and exhibits anisotropic response to stimuli of heating and light irradiation. Furthermore, hydrogels with sophisticated 3D configurations could be obtained by multi-step magnetic orientation and photolithographic polymerization, which could realize versatile locomotion.

In addition, magnetic particles could be used as actuators for the alignment of the polymer chains that obtained mechanically anisotropic hydrogels. Inducing by the magnetic field, the magnetic assembly drag not only the magnetic nanoparticles, but also the polymer attached to nanoparticles because of attraction between them. Gila-Vilchez et al. (2019) applied this strategy to design and three different anisotropic magnetic hydrogels such as magnetic Fmoc-diphenylalanine peptide hydrogels, magnetic fibrin hydrogels and magnetic alginate hydrogel. The microstructural arrangement of the hydrogel network was obtained medicated by magnetic field induced assembly of magnetic nanoparticles, which lead to stronger mechanical properties than isotropic magnetic hydrogels. Similarly, Contreras-Montoya et al. (2018) also reported the synthesis and structural characterization of Fmoc-diphenylalanine peptide supramolecular hydrogels with ordered magnetic structure. The column-like aggregates of magnetic nanoparticles were engulfed and fixed by the gel-forming peptides without change of the hydrogel pore size. This magnetic hydrogel exhibited strongly enhanced mechanical strength and improved diffusion of a small solute through it, providing promising application in drug delivery and tissue engineering. More interestingly, Chen et al. (2022b) constructed ordered structure inside the magnetic polyvinyl alcohol/polyacrylic acid hydrogel by magnetic-field-induced assembly, freezing-thawing and annealing process, showing anisotropic friction performance. Not only the oriented structure of magnetic materials could be obtained, but also ordered and dense structure of polymer network could been seen in the hydrogel. Thus, the hydrogel exhibited outstanding mechanical properties including high tensile strength, toughness and compressive strength. And the aligned nanoparticles strongly influenced the hydrogels in terms of their shear behaviors and viscoelasticity, resulting in anisotropic tribological properties. Very interestingly, a recent research reported a chain-like ferrimagnetic nano-assemblies within hydrogels *in situ* (Sturm et al., 2019). The authors used thermoreversible gelatin hydrogel matrix as a template structure to synthetic ferrimagnetic magnetite chains, which inspired by chains of ferrimagnetic nanocrystals from magnetotactic bacteria. Gelatin hydrogel loaded with Fe (II)-ions was placed into the NO^3−^ and OH^−^ solution, initiating the formation of ferrimagnetic nanocrystals. Those ferrimagnetic particles inside the hydrogel arranged in chains due to dipolar interactions, which was magnetic anisotropy. The bioinspired strategy showed an easy green synthesis route to generate magnetically anisotropic material. However, it remained challenging in producing perfect defect-free single crystals in the synthesis.

Recently, photonic crystal structures that can change colors in the visible light spectrum were constructed, which produced by 1D assembly of colloidal magnetic nanoparticles (He et al., 2012). However, the structural colors in liquid media are unstable and would disappear immediately when the magnetic field is removed. Therefore, hydrogel matrix have been widely used to prepare and control the assembly structure of colloidal magnetic nanoparticles, resulting in stably display colors (Hu et al., 2015b). And combined with lithographical techniques, the multicolored photonic crystal patterns could be obtained within the hydrogels (Wang et al., 2022b). Hydrogel matrix could undergo a volume change in response to stimulation, changing the lattice constant of photonic crystals which resulted in the color change (Shi et al., 2020a). In order to realize on-demand assembly with high spatial and temporal resolution, DNA supramolecular hydrogels were used to control gelation and degelation process recently (Dong et al., 2021).

The magnetic-field-induced assembly assisted fabrication of magnetic hydrogels with ordered structures brings promising opportunities for design of biomedical devices or soft robots.

### Microfluidic techniques

Microfluidic technique is a powerful platform for hydrogel microparticle generation (Cai et al., 2021). Different from bulk hydrogels, hydrogels with smaller size allow minimally invasive injection which are more suitable for biomedical applications (Daly et al., 2020). Using a microfluidic chip, fluids was pumped through narrow microchannels at low pressures, showing laminar flow resulting from low Reynolds number. And deriving from the dynamic of multiple fluids, droplets can be generate which can form hydrogel microparticle after polymerization. By precise flow control of fluids in microchannel, not only the number and size of microparticle but also the morphology and structure of microparticle could be precisely controlled (Fu et al., 2018). Therefore, microfluidic technique have attracted increasing scientific attention on preparing magnetic hydrogel with order structure.

Microfluidic-emulsion techniques use immiscible oil and aqueous hydrogel precursor solutions to generate droplets in the microchannel that can then be crosslinked into microhydrogels (Daly et al., 2020). By carefully controlled the microchannels and fluidics, the hydrogel can be imparted tailored components, structures and properties. A variety of magnetic hydrogels have been developed with well-defined shapes or microstructures such as Janus, fiber-like, peanut-like, multi-compartment hydrogel microparticles. For example, Liu and Nisisako (2022) prepared magnetic Janus Ca-alginate hydrogel microparticles *via* a microfluidic emulsion-based external gelation method. The two compartments showed clear boundaries and their volume fraction could be readily adjusted. Wu et al. (2018) developed a co-axial microfluidic system to fabricate peanut-like magnetic hydrogel microparticles with their inner cores arranged controllably. With magnetic nanoparticles encapsulated inside hydrogel, those micro particles exhibited extraordinary controllable ability under a rotating magnetic field. Those anisotropic hydrogel microparticles discussed above showed magnetic anisotropy, allowing self-assembly, rotation, and accumulation under a magnetic field. Such magnetic functionality is promising for soft actuation and magnetic separation. Zhao et al. (2012) reported barcode hydrogel particles containing photonic crystals and magnetic nanoparticles. Based on photo-curable microfluidic multiple emulsion templates, multiple spherical photonic crystal and magnetic cores were fixed inside polyethylene glycol (PEG) hydrogel shells with ordered structure. The presence of magnetism in the barcode hydrogels confers their controllable rotation and aggregation under magnetic fields. Inspired by the multicompartment structures of cellular architectures, Tan et al. (2017) fabricated structured hydrogel particles with multiple compartments. The distinguished compartments inside hydrogel particles was assembled *via* microfluidic-emulsion techniques. The authors loaded glucose oxidase and magnetic nanoparticles into defined compartments, allowing occurrence of a glucose-triggered, incompatible, multistep tandem reaction. This system exhibited great advantages in mimicking the biological process.

Microfluidic electrospraying is also a powerful technique for generation microhydrogels with ordered structure (Wang et al., 2018). During the electrospray process, the precursor solution was injected and extruded through microfluidic channels. Then, the fluid was broken up into droplets under the high electric field and sprayed into collecting bath for further crosslink (Gansau et al., 2018). Thomas et al. (2018) prepared calcium alginate Janus microspheres with mesenchymal stem cells in one compartment and magnetic nanoparticles in the other compartment. The encapsulated cell could be segregated from foreign material, showing excellent viability. In addition, the magnetic Janus hydrogels allowed precise magnetic manipulation, such as linear and rotational movement. However, types of hydrogel and their crosslink method used in microfluidic electrospraying are usually limited, and there remains obstacles in control of monodispersity and high uniformity. Unlike microfluidic electrospraying that control the flow of fluid based on strict microfluidic devices, Kang et al. (2019) developed a modular micronozzle system driven under centrifugal force to prepare alginate microspheres with various structures and sizes. With just a centrifuge and easy combined micronozzle system, magnetic Janus hydrogel particles could be readily generated. Although achieving hydrogel particles with low dispersity index is challenging, it still presents a fast, reproducible and scalable manner that is particularly useful for the large-scale synthesis.

**FIGURE 4:**
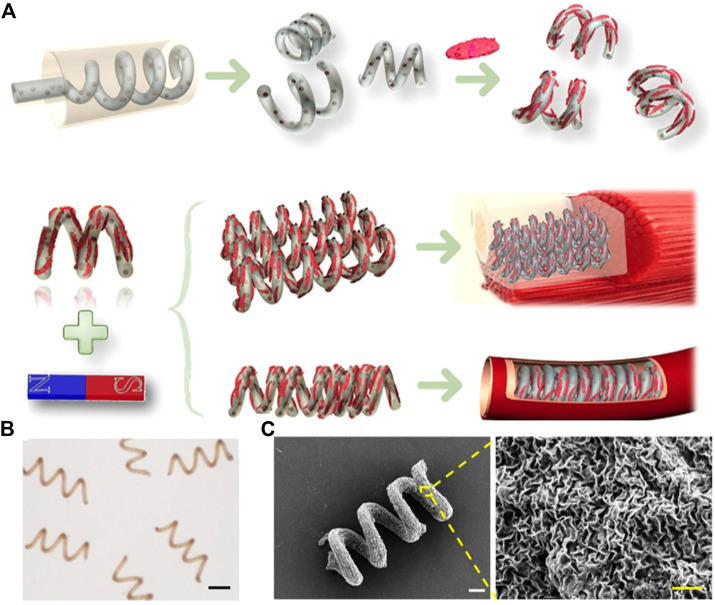
Magnetic helical micromotors prepared with microfluidic techniques. **(A)** Schematic illustrations of the fabrication of the helical micromotors and their potential applications. **(B)** Photograph of the helical micromotors. Scale bar = 300 µm. **(C)** SEM images of the helical micromotor. White scale bar = 20 μm, Yellow scale bar = 2 µm. (Reproduced with permission from (Yu et al., 2020) Copyright 2020 American Chemical Society).

Recently, an emerging magnetic hydrogel with fiber structure (spring shape) have attracted great scientific interest due to their unique shape and mobility. After production of microfiber hydrogels *via* microfluidics system, homogeneous anisotropic microgels with various morphologies could be obtained by controllable destruction (Cai et al., 2021). Zhao and coworkers first generated microfibers with anisotropic multicompartment by microfluidic techniques (Yu et al., 2017a). With the inner Na-alginate liquid flow was injected into a CaCl_2_ solution, the fluid started to spin and crosslink, leading the formation of anisotropic helical microfiber. These hydrogel microfibers containing magnetic nanoparticles could be stretched by a magnet and recover to original shape when the magnet was moved away, which can be used as microspring with magnetic responsivity. In another work, they integrated flow lithography into microfluidic systems to precisely control the generation of helical microparticles, forming unique shapes such as Janus, triplex, and core–shell cross-sectional structures (Yu et al., 2017b). The helical microparticles containing magnetic nanoparticles could display tumbling motion and translational corkscrew motion under a 3D rotating magnetic field. Based on their precise work, they presented a magnetically helical microhydrogel as dynamic cell microcarriers (Figure 4) (Yu et al., 2020). The helical microhydrogel could migrate, aggregate, and assemble into complex geometrical structures by magnetic actuation, exhibiting the ability to shape a cell stack or a cell tube. It should be mentioned here that many efforts have been made to helical shaped magnetic hydrogel with simplified devices. For example, Yoshida and Onoe (2017) fabricate hydrogel microsprings just using basic capillaries and syringe pumps. In this work, the magnetic nanoparticle-encapsulating microspring exhibited magnetic responsiveness and typical characteristics of a mechanical spring. Liang et al. (2019) prepared magnetic hollow microfibers with non-coaxial microfluidic device. By changing the distance between the side-by-side nozzles and liquid level, hollow hydrogel microfibers with diverse geometries (such as single- and double-helical springs, necklaces and ladders) were generated. The hollow structure was formed by a series of chemical reactions, providing unique perfusable channels. More interestingly, when loading magnetic particles in only one end of the magnetic microfiber, it could generate jumping motions by magnetic actuation because of the asymmetric magnetic interaction. Wang et al. (2021) manufactured helical magnetic poly (vinyl alcohol) (PVA) hydrogel by extruding precursor solution and melamine (crosslinker) onto a rotating syringe needle. The helix pitch and diameter allowed to be well regulated by adjusting needle diameter and rotating speed. This helical magnetic PVA hydrogel can be used for precisive navigation and direct T cell chemotaxis by loading chemokine.

The good control over the gel-formation process is the main advantage of microfluidic techniques, however they are still limited to low throughput and complex devices. In addition, researchers should pay more attention on the choose of hydrogel matrix and chemicals used during preparation in order to improve biocompatibility.

### 3D printing

Additive manufacturing (i.e., 3D printing) is a practice of building complex functional 3D structures in stacked form (Ge et al., 2021). Given digital design from computer, 3D printing technology allows directly creating products with intricate internal structure on micrometer to centimeter sizes (Yang et al., 2022a). With the magnetic nanoparticles dispersed in the hydrogel ink, 3D printing can enable the formation of magnetic ordered structure. Several printing technologies have recently been developed for the magnetic hydrogel, such as direct ink writing (DIW), inkjet printing (IJP), digital light processing (DLP) and two-photon polymerization (TPP) (Zhang et al., 2021). The detailed development of various 3D printing technologies have already been summarized in several recent reviews (Malekmohammadi et al., 2021; Xue et al., 2021a; Zhang et al., 2021; Ganguly and Margel, 2022). Programmable patterning of the hydrogels results in the creation of macroscopically anisotropic magnetic material. The resulting magnetic hydrogels tend to exhibit finer structures and better functionality (Podstawczyk et al., 2020; Ajiteru et al., 2021). It is worth mentioning that 3D printing processes can be integrated with magnetic field induced assembly, and parallelized (Wang et al., 2017; Zhang et al., 2021). The magnetic field can be imposed to the flowing ink or the printing location to tune the orientation and spatial arrangement of magnetic nanoparticles. Although owning the advantages mentioned above, 3D printing still remain some challenges, such as relatively low throughput and expensive devices.

IJP is a powerful method for magnetic hydrogel processing resulting from its relatively high automaticity. The ink droplet is extruded from the print head and ejected onto the substrate with ordered patterns or structures. To avoid the clogging during printing, magnetic ink usually selected polymer solution with low viscosity and magnetic particles with nano size (Gao et al., 2021). In addition, IJP technique is lack in printing accuracy, limiting its construction of complex and ordered structures. DIW is a layer-by-layer assembly methodology that extruded print materials from dies to control topologies (Chen et al., 2019; Ge et al., 2021). When using magnetic materials, magnetization distributions can be readily tuned during the printing process. However, the solidification of hydrogel inks was hard to control, leading to the fidelity of printed structure and clogging of the nozzle (Chen et al., 2019; Wulle et al., 2022). In recent years, multimaterial direct ink printing (4D direct ink printing) which employs an extra time dimension has emerged as a fast and straightforward technique to generate various hydrogel robots. Simińska-Stanny et al. (2022) developed a graded and patterned 4D-printed magnetic hydrogels showing spatially anisotropic response to magnetic fields. Before printing, the magnetic inks were subjected to the magnetic field, resulting in the arrangement of incorporated magnetic nanoparticles.

**FIGURE 5:**
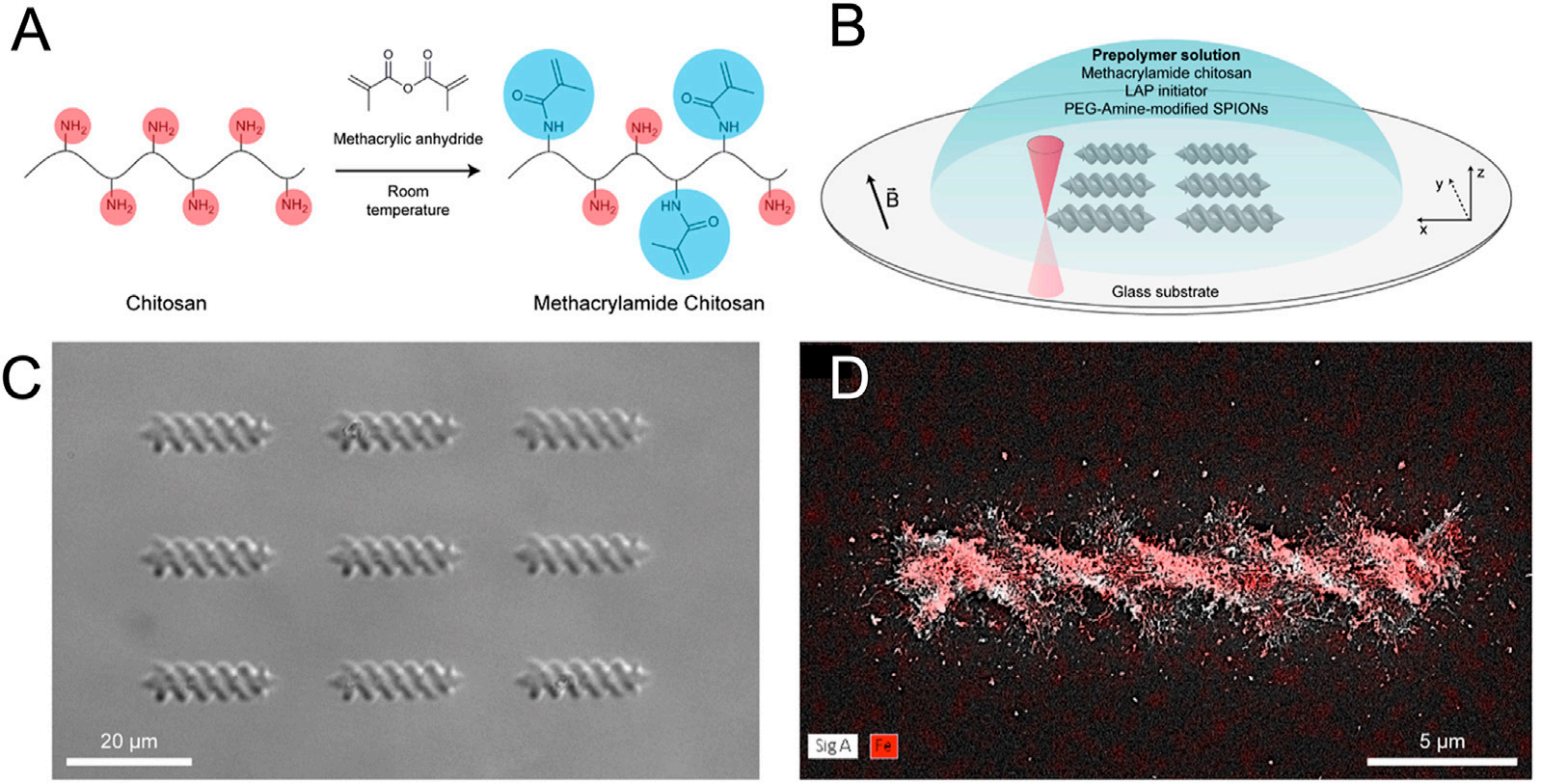
Synthesis and fabrication processes of magnetic microswimmers. **(A)** Synthesis of the photo-cross-linkable methacrylamide chitosan. **(B)** 3D printing of the microswimmers using two-photon direct laser writing technique. **(C)** Microscopy image of 3D-printed microswimmers. **(D)** Energy-dispersive X-ray spectroscopy elemental mapping showing the presence of iron atoms (red color) in the microswimmers. [Reproduced with permission from (Bozuyuk et al., 2018) Copyright 2018 American Chemical Society].

Recently, photo-polymerization that uses specific light source to solidify light-sensitive liquid polymers achieved great interest due to its high resolution (Zhang et al., 2021). To obtain complex or fine 3D structures, multiphoton light sources have been used. TPP was able to produce magnetic hydrogels with features defined at the sub-micrometre scale (Fiedor and Ortyl, 2020). For instance, Bozuyuk et al. (2018) fabricated the magnetic chitosan-based microswimmers with helical structure by two-photon 3D printing, as shown in Figure 5. Due to the introduction of magnetic nanoparticles and photocleavable linker, the magnetic microswimmers could be well controlled under rotating magnetic fields and realize light-triggered release on-demand release drugs, exhibiting significant potential in drug delivery. Cabanach et al. (2020) developed fully zwitterionic non-immunogenic photoresists to construct magnetic hydrogel microrobots with non-immunogenic properties by TPP 3D printing. Combined with magnetic nanoparticles, the helical hydrogel microrobots allowed microscale actuation *via* magnetic torque. However, high concentration of magnetic particles was not allowed because of colloidal stability problems and magnetic nanoparticle induced absorption.

3D printing provides an unprecedented, excellent tool to create magnetic hydrogels with ordered structures and additional functions. It is critical in future attempts to improve the printing accuracy, design customized structures and develop biocompatible inks.

## Biomedical applications of magnetic hydrogels with ordered structure

Imparting magnetically responsive materials to the hydrogels with order structure, magnetic hydrogels can respond to stimuli and showing macroscopic behaviours, such as magneto-motion and magneto-thermal effect (Li et al., 2013). Such unique ordered structure is practically useful for regulating magnetic effects and developing novel functionality. The special properties and similarity to biological soft tissue make magnetic ordered hydrogels achieve great process in various biomedical applications, such as soft robotics, drug delivery, tissue engineering and biosensing (Shi et al., 2020a; Li et al., 2021). In this section, we discuss the biomedical applications of magnetic hydrogels with order structure and their corresponding performances.

### Soft robotics

The soft robotics exhibit flexible deformability and mobility in response to the external stimuli, resulting in widely applications in drug delivery and minimally invasive operation (Yarali et al., 2022). Hydrogels own excellent biocompatibility and can be flexibly designed as soft robotics with expected functions, which attract great attention (Zhou et al., 2021). Moreover, hydrogel materials have degradation abilities, showing promising in clinical applications compared with those non-degradable materials (Yang et al., 2022a). Different from other stimuli-responsive hydrogels, magnetic hydrogels can realize remote noncontact control and deep tissue penetration, which is critical for applications in physiological environment. To achieve complex manipulation and effective actuation, imparting ordered structure to magnetic hydrogels is a promising route.

Magnetic actuation is the basic function of soft magnetic robotics. Under the wireless magnetic field, the magnetic robots could be actuated actively and positioned to the target area precisely. Particularly, magnetic robots with structure such as helical shape could be actuated by magnetic torque, showing a more effective route for magnetic navigation. For example, Bozuyuk et al. (2018) fabricated helical microswimmers by 3D printing. Under a rotating magnetic field, the helical microswimmers could be operated in the low Reynolds number regime with great controllability. The biodegradation and light-triggered drug release capability could be imparted to microswimmers by chemical modification without affecting magnetic actuation capabilities. Moreover, the microswimmers was biodegradable and biocompatible.

**FIGURE 6:**
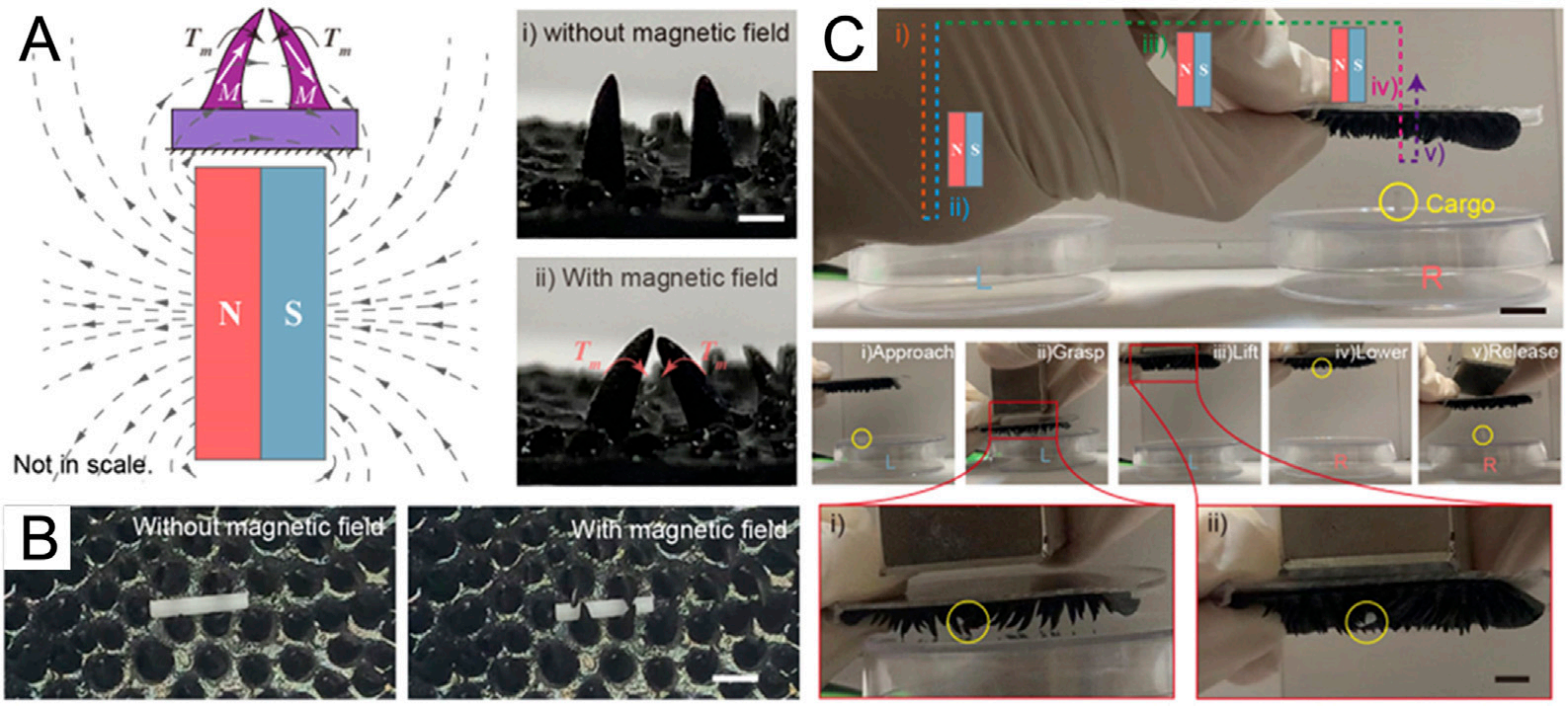
Grasping function of the claw-inspired hydrogels. **(A)** Motions of the legs with a permanent magnet. Due to the shape of the legs, the magnetic moment could cause the left and right legs to move towards each other, like grasping operation of claws. Scale bar = 1.5 mm. **(B)** Using magnetic fields to control the operation of grasping and releasing Φ1×5 mm cylinder. Scale bar = 2 mm. **(C)** Transportability test for grasping cylinder. Scale bar = 10 mm and 5 mm. [Reproduced with permission from (Yang et al., 2022b) Copyright 2022 American Chemical Society].

Magnetic shape morphing is another function of magnetic soft hydrogels, drawing more and more attention. Magnetic hydrogels with anisotropic structure can change into desired shapes by swelling or mechanical stimulation. For example, Tognato et al. (2018) developed a starfish-shaped magnetic soft robot with 3D printing technique. Due to the anisotropic distribution of the magnetic nanoparticles in their fins, the starfish-shaped robots could perform swimming, wrapping and flapping motions by applying suitable magnetic fields. In addition, based on the volume change of thermosensitive hydrogel, the magnetothermal effect may also be used to actuate the motion of soft hydrogels. For example, (Tang et al., 2019), applied an alternating magnetic field to actuate magnetic poly (N-isopropylacrylamide) hydrogels which can be heated and undergo volume shrinkage under the magnetic stimulation. Under the alternating magnetic field, the arrangement of the magnetic hydrogel pattern on 1D elastomer strips or 2D elastomer sheets could transformed into a variety of 2D and 3D shapes, such as truss, tube, helix and heart-shape etc. Moreover, magnetic hydrogels with hinges could be folded into origami structures remotely. With the development of bionic science, more soft robots based on magnetic hydrogel were reported inspired by nature. For example, (Yang et al., 2022b), prepared a soft millirobot based on a magnetic hydrogel with a multilegged structure. The multilegged structure was inspired from the insect claw-like legged structure, which could perform motions, grasping and cargo transportation (Figure 6). To explore the locomotive ability of hydrogel robots in highly viscous Newtonian fluids, (Bhattacharjee et al., 2022), designed a bacteria-inspired soft robot. Similar to swimming microorganisms, the tapered rod-like soft robots could perform rolling and swimming locomotion in viscous Newtonian fluids by a uniform rotating magnetic field.

With the investigation of relations between structure, locomotion and function in the future, hydrogel robots may inspire more biomedical applications. However, their mechanical properties might not match complicated biological tissues at present. Moreover, the balance between stability and degradability *in vivo* should be investigated according to different requirements.

### Drug delivery

Hydrogels have long been regarded as ideal drug delivery carries, owing to their efficient drug encapsulation, precise drug transport, and predictable drug release (Bordbar-Khiabani and Gasik, 2022). The porous network and adjustable degradation of hydrogels allow drugs release sustainably (Daly et al., 2020; Ali et al., 2022). However, those passive drug release methods are lack of efficiency and regulation. Magnetic hydrogels with ordered structure can carry drug to targeted lesions and control drug release under the magnetic field, overcoming limitations of traditional hydrogels. And magnet-controlled drug delivery allows remote operation which is necessary for applications *in vivo*.

Many researches have proved that the drug release from hydrogels could be controlled by magnetic field (Zhang et al., 2016a; Li et al., 2021). The magnetic nanoparticles tend to move in the polymer network and change the porosity under the external magnetic stimulation, thus achieving the control of drug release (Lin and Metters, 2006). The magneto-thermal effect is another mechanism of magneto-controlled drug release (Lin and Metters, 2006). Under a high-frequency alternating magnetic field, the magnetic nanoparticles generate heat due to the Neel and Brownian relaxations. The resulting heat can speed up the molecular motion that promote the diffusion and release of drug. The degradation of polymer matrix can also been tuned by magneto-thermal effect, leading the drug release (Li and Mooney, 2016; Zhang et al., 2016a). For example, Hu et al. (2015a) used magnetic field to control drug release from magnetic hydrogels with anisotropic structure. Owing to the anisotropic assembly of magnetic nanoparticles, the thermogenesis could be controlled by changing the external alternating magnetic field’s direction. By changing the angle between the assembled chains and the magnetic field, the thermogenesis changed which directly influenced the released amount of doxorubicin from hydrogels. In addition, the magnetic hydrogel with aligned structure showed improved doxorubicin release compared with disordered magnetic hydrogel. Furthermore, Monks et al. (2021) used extrusion-based 3D printing to prepare patterned hydrogels based on ink composed of Pluronic F127 and magnetic nanoflowers. The hyperthermic response could be controlled by altering particle concentration, feature volume and feature surface area. The authors demonstrated that the spatial distribution of heat could be well manipulated by spatial patterning. As the magnetic hydrogels with ordered pattern could generate temperature gradients, spatiotemporally-controlled release of drug was possible. It is worth noting that magnetic microhydrogels with multi compartments could be used to load different drugs, avoiding cross-pollution. Chen et al. (2022c) fabricate a magnetic core/shell hydrogels by microfluidic techniques for composite drugs delivery. The authors loaded two drugs, hydrophobic camptothecin and hydrophilic doxorubicin in the core and shell layers of hydrogel particle. And the magnetic nanoparticles loaded in the shell layer could generate thermal effect to control the release of encapsulated drugs. In the future, imparting magnetic compartment to realize independent release of selected drug would be interesting.

The magnetic hydrogels with order structure achieve many developments in drug delivery, but some concerns and challenges remain in term of clinical applications. For instance, magneto-controlled drug release commonly used high concentration of magnetic nanoparticles which might do damage to bodies. Furthermore, the long-term residency of magnetic nanoparticles and hydrogels *in vivo* are not suitable, leading to the requirement of controllable biocompatibility and degradability.

### Tissue engineering

Owing to their native extracellular matrix-like features, magnetic hydrogels have been proved to be promising biomaterials as tissue engineering templates (Seliktar, 2012; Wang et al., 2022a). Many biological tissues present an specific organization of ordered structures, which is important for physiological functions (Shi et al., 2020a). Magnetic hydrogels with ordered structure can provide an template for directional growth of cells and control their behavior, which have attracted extensive researches (Liu et al., 2019; Liu et al., 2020; Park and Kim, 2020).

**FIGURE 7:**
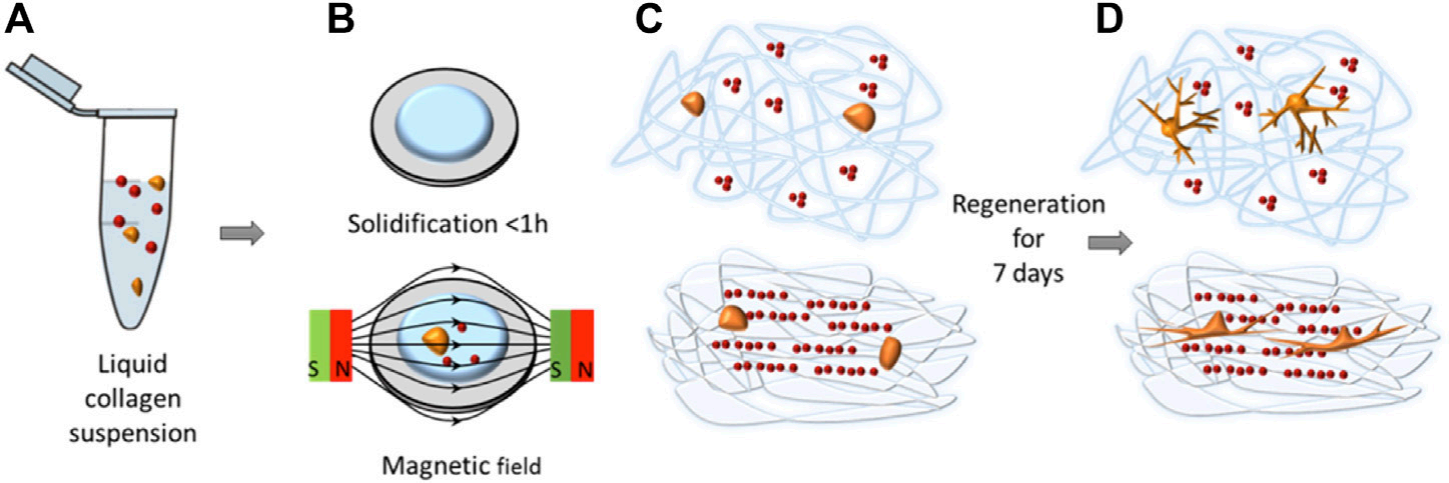
Schematic illustrations of magnetic hydrogels with alignment structure for guiding neuronal regeneration. **(A)** Collagen precursor solution containing neurons (orange) and magnetic nanoparticles (red). **(B)** Solidification of the solution with/without a magnetic field. **(C)** Magnetic field can induce the linear alignment of magnetic nanoparticles and collagen fibers. **(D)** Magnetic nanoparticle chains and fibers can guide the growth of neurons as topographical cues. (Reproduced with permission from (Antman-Passig and Shefi, 2016) Copyright 2016 American Chemical Society).

As magnetic hydrogels with order structure are similar to the native mechanical microenvironment *in vivo*, they are more suitable for cell culture than traditional hydrogels. For example, Hu et al. (2016) utilized sliced magnetic hydrogel with anisotropic architecture as 3D cell culture platform. The magnetic nanoparticles formed chain-like assembly structure within the polyacrylamide hydrogel, enhancing interactions between cells. Even using a classic 3D cell culture model, cells could form multicellular spheroids spontaneously rather than loose aggregates. For tissue regeneration, magnetic hydrogels with ordered structure were also used to mimetic native tissues and trigger their natural reparative process, which have been applied in nerve, skin, cartilage and muscles (Shi et al., 2020b; Liu et al., 2020). For example, Antman-Passig and Shefi (2016) developed magnetic collagen hydrogels with orienting structure to direct neuronal regeneration (Figure 7). The alignment process could be controlled dynamically and remotely *in situ*, leading to the alignment of magnetic nanoparticles and even collagen fibers. As scaffolds for neuronal regeneration, the hydrogels can direct neuronal growth that induce neurons to form cooriented morphology. By altering the magnetic field, hydrogels with different microstructure orientations could be obtained for various tissue engineering. Shi et al. (2020b) prepared silica rods coated with magnetic nanoparticles to impart orientational order with collagen hydrogels under a magnetic field. These highly anisotropic nanorods could be readily oriented by a weak magnetic field, and thus influence the growth of normal human dermal fibroblasts. A recent work applied magnetic collagen-based hydrogels with oriented structure to direct the growth of human adipose stem cells (hASCs) (Wright et al., 2022). Actin filaments of hASCs oriented along to the aligned collagen fibers and MNPs. The constructs could emulate physiologically and pathologically tissues, providing a promising method for tendon repair. In another work, Tognato et al. (2022) prepared anisotropic collagen-based substrates to guide the growth of human mesenchymal stem cells.

Injectable magnetic hydrogels show great potential for tissue regeneration duo to their minimal invasion. For example, Omidinia-Anarkoli et al. (2017) reported an injectable hydrogel with oriented magnetic fibers to guide the growth of nerve cells. Short fibers containing MNPs were dispersed inside the precursor solution, then anisotropic structure could be formed *in situ* within a magnetic field before hydrogel crosslinking. Different from hydrogels without oriented structure, those ordered hydrogels were able to guide the growth and extension of fibroblasts and nerve cells, providing a minimal invasive route for complex tissues regeneration. In a recent research, the types of hydrogel matrix and its stiffness, and surface biomodifications were also proved to affect the cell responses (Babu et al., 2022). Micro hydrogel with small size is another method to realize minimal invasion and remote control. With a straightforward microfluidic spinning and coiling approach, Yu et al. (2020)fabricated magnetically helical microhydrogels as dynamic cell carriers. These magnetic microhydrogels with high biocompatibility showed great performance in cell adhesion and cultivation. Due to the magnetic nanoparticles encapsulated in helical structure, the microhydrogels allowed controllable manipulation of motion under the magnetic field, which could be used to form a cell tube or stack.

Although magnetic hydrogels with ordered structure showed great advantages on the regeneration of anisotropic tissues, there remain challenges such as the mechanical mismatch of hydrogel matrices and the cytotoxicity of magnetic materials. It is worth noting that magnetic materials have been used in neural modulation *via* magnetic stimulation. A recent work reported a magnetic hyaluronic hydrogel that could realize noninvasive neuromodulation by magnetomechanical stimulation (Tay et al., 2018). The magnetic microparticles were evenly distributed inside the hydrogels which could mechanically stimulate somas and neurites together. Considering the nervous tissues commonly exhibit anisotropic architecture, we expect that magnetic hydrogels with ordered structure might be more promising for neuromodulation.

### Biosensing

The magnetic colloidal nanoparticles were able to form photonic crystals by magnetic-field-induced assembly (Shi et al., 2020a). Immobilizing photonic crystals inside stimuli-responsive hydrogels allows it to exhibit color changes that respond to the stimulation and environment (Tang and Chen, 2020; Shen et al., 2021). Therefore, structural color hydrogels using magnetic colloidal nanoparticles have attract many researches in terms of biosensing.

**FIGURE 8:**
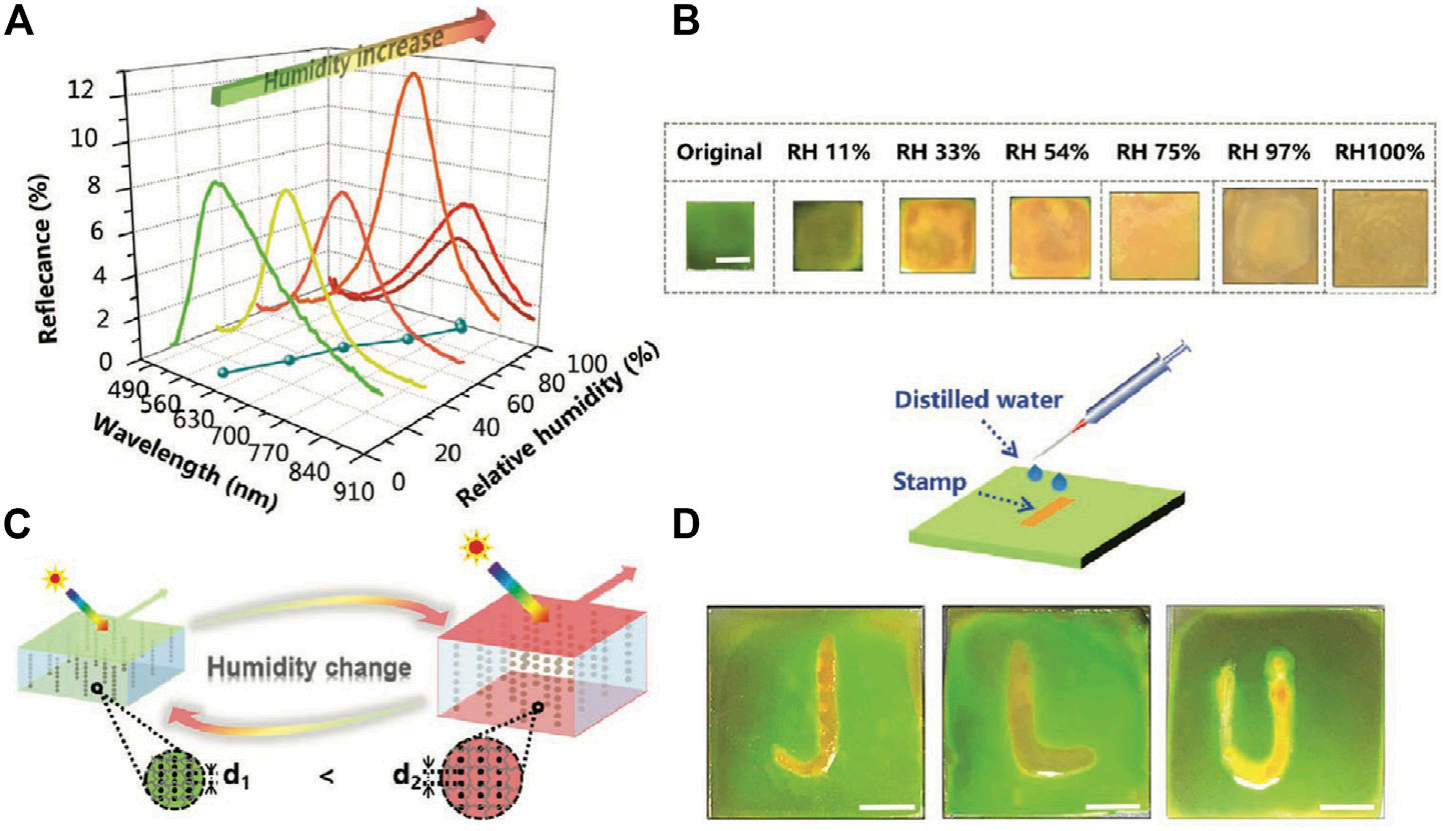
Structured color hydrogel film with response to humidity. **(A)** Reflection spectra. **(B)** Photographs of the hydrogel film under different relative humidity. **(C)** Schematic diagram of the discoloration mechanism of the hydrogel film. **(D)** Schematic diagram of writing letters with distilled water on the photonic display tablets and reasonably accurate digital photos. Scale bar = 0.5 cm. [Reproduced with permission from (Xue et al., 2021a) Copyright 2021 American Chemical Society].

For example, Shang et al. (2020) fabricated thermochromic photonic fiber hydrogels, exhibiting chromatic sensitivity to temperatures. The colloidal nanocrystal clusters (carbon-encapsulated Fe_3_O_4_) were formed into chain-like assemblies in the presence of an external magnetic field. Additionally, 1D chain-like structures were fixed using poly (N-isopropylacrylamide), a thermo-responsive hydrogel. Temperature-dependent tuning of the spacing between magnetic nanoparticles in chain-like structures might provide controllable and reversible structural colors. Xue et al. (2021b) prepared a structured color hydrogel film with adaptive discoloration in response to environmental change (Figure 8). The volume of the hydrogel film altered in response to stimuli such as humidity, ion concentration, and mechanical stress, which regulated the lattice spacing in the chain of magnetic nanoparticles and spontaneous color shifts. The photonic is appropriate for biosensing and diagnostics since color change is a simple and straightforward method of feedback. Recently, Wang et al. (2022b) manufactured a variety of structural color hydrogel patterns through magnetically induced regional crosslink of polymer. The programmable pattern could be obtained by control the magnetic-field-induced assembly and mask-assisted UV polymerization. The change of structural color was dynamic and reversible with the changes of humidity and light, showing powerful ability for biosensing.

**TABLE 1:** The preparation strategies of magnetic hydrogels with ordered structure.

Strategies	Details	Ordered structure	Properties	References
Magnetic-field-induced assembly	Magnetostatic field	Linear assembly	Anisotropic magnetothermal effect	Hu et al. (2015a), Rincon-Iglesias et al. (2022)
	Rotating magnetic field	Disk-like assembly	Anisotropic magnetothermal effect	Fan et al. (2018)
	Magnetostatic field	Linear assembly	Enhanced mechanical property; Alignment of polymer chains	Contreras-Montoya et al. (2018), Gila-Vilchez et al. (2019)
	Magnetostatic field	Linear assemblies	Structural colors	Hu et al. (2015b), Xue et al. (2021a), Wang et al. (2022b)
Microfluidic techniques	Microfluidic-emulsion	Janus; multicompartment; fiber (helical shape)	Anisotropic properties, multi-functional	Zhao et al. (2012), Yu et al. (2017a), Wu et al. (2018), Liu and Nisisako (2022)
	Microfluidic electrospraying	Janus; multicompartment	Anisotropic properties; multi-functional	Thomas et al. (2018)
3D printing	Inkjet printing	Customed pattern	Anisotropic properties	Gao et al. (2021)
	Direct ink writing	Customed pattern	Anisotropic properties	Chen et al. (2019)
	4D Printing	Customed pattern	Anisotropic properties	Simińska-Stanny et al. (2022)
	Two-photon 3D printing	Customed pattern	Anisotropic properties; High resolution	Bozuyuk et al. (2018), Cabanach et al. (2020)

In the future, it is very important to investigate mechanism of color changes and improve the sensitivity and accuracy of sensing. The decoding process usually relies on detecting changes of shape and color *via* external equipment, thus the convenience and consistency of decoding should also be taken into consideration.

## Conclusion and prospects

Hydrogels have attracted extensive researches because of their high hydrophilicity, good biocompatibility and adjustable degradability. Different from traditional hydrogels, magnetic hydrogels with ordered structure have exhibited immensely potential because of their superior functional performances. In this review, we have summarized the fabrication of magnetic hydrogels with ordered structure and their physical properties. The ordered structure could bring anisotropic properties and unique functionalities to hydrogels, making it more intelligent. Finally, we have reviewed the biomedical applications of ordered structural magnetic hydrogels.

Although the development of magnetic hydrogels with ordered structure has brought significant advances to biomedical researches, there remain some challenges that needed to be resolved. Magnetic-field-induced assembly is a simple and flexible method, while it is unsuitable for construction of complex structure inside hydrogels. Although microfluidic techniques and 3D printing can conveniently generate particular structure with high uniformity, they usually rely on complex system and strict technical parameters, resulting in the incapability of mass production. For mass fabrication of ordered structural hydrogels with high spatial resolution, it is necessary to develop advanced manufacturing technology. The simpler and higher-precision manufacturing technology will strongly promote their biomedical applications. In addition, it will be important to investigate the magnetic property of ordered structure and designs of bionic architecture in order to develop extensive functionality and applicability for hydrogels. Although many magnetic hydrogels with various ordered structures have been made, their physical and chemical properties (especially the collective magnetic property) are not fully revealed. For example, the research on magneto-thermal effect of magnetic ordered hydrogels and their biomedical applications are relatively elementary. It is expected that exploration on the magnetic properties of magnetic ordered hydrogels will benefit the comprehension of magnetic biological effects. Due to the complex environment and unique structure of tissues, it is necessary to delicately design suitable hydrogel matrix and magnetic structure for different applications. We believe that with the cooperation of interdisciplinary research, the biomedical applications of magnetic ordered hydrogels will take things to the next level, especially in tissue engineering. Last but not least, biosafety of magnetic hydrogels deserves important consideration, thus requiring comprehensive studies on their biocompatibility and biodegradability *in vivo*. Not only hydrogel polymers but also magnetic nanoparticles should be studied to understand their long-term metabolism and toxicology. As several hydrogel products and magnetic nanoparticles have been approved by FDA to applied in clinic, we anticipate that the magnetic ordered hydrogels made of FDA-approved materials will soon make a real impact in biomedical fields and even clinics (Bobo et al., 2016; Thakar et al., 2019; Li W. et al., 2022). To expand biomedical applications, it is meaningful to impart multiple responsiveness and more functionalities to the magnetic hydrogels with ordered structure. Thus, those smart hydrogels can simultaneously perform a variety of biomedical tasks which is beneficial and indispensable in practical applications.

We expect this review could inspire more scientists from biomedical engineering, material science and intelligent manufacturing to impelling the fabrication of magnetic hydrogels with ordered structure and boost their biomedical applications.
